# Editorial: Genetics of pediatric immune-mediated diseases

**DOI:** 10.3389/fgene.2025.1771063

**Published:** 2026-01-19

**Authors:** Sara Manti, Beatriz Elena Marciano

**Affiliations:** 1 Pediatric Unit, Department of Human Pathology in Adult and Developmental Age “Gaetano Barresi”, University of Messina, Messina, Italy; 2 Laboratory of Clinical Immunology and Microbiology, National Institute of Allergy and Infectious Diseases, National Cancer Institute, National Institutes of Health, Bethesda, MD, United States

**Keywords:** disease, genetic, genotype, immune system, pediatric, phenotype

Pediatric immune-mediated diseases are a diverse group of conditions in which a child’s immune system mistakenly targets and damages its own tissues, leading to chronic inflammation and other serious health issues. Volume I of our Research Topic, including four diverse studies—from case reports to large-scale genetic analysis—collectively provides a comprehensive, contemporary update on the genetics of pediatric immune-mediated diseases.

Wang et al., in their case report, first described, through a retrospective analysis, a case of X-SCID caused by a novel *de novo* IL2RG mutation, thereby expanding the known mutation spectrum and highlighting its immunological and diagnostic relevance. The patient’s presentation (chronic diarrhoea, recurrent fever, and anemia, with poor response to antibiotic treatment) and laboratory findings -showing reduced T lymphocytes and NK cells, decreased levels of immunoglobulins (IgG, IgA, IgE) and T. marneffei infection - underline the importance of diagnostic value of integrating phenotypic and molecular analyses in patients with suspected Inborn Errors of Immunity.

In line with this, Li et al. described a case of progressive pseudorheumatoid dysplasia (PPRD) involving a novel cellular communication network factor 6 (CCN6) gene mutation (c. 802T>C and c.624dup) and sacroiliac and hip arthritis, including bone marrow oedema and joint effusion. PPRD is a rare, autosomal recessive arthropathy, often misdiagnosed as juvenile rheumatoid arthritis (JIA), spondyloarthritis (SpA), and other inflammatory diseases clinically. Although it is defined as a noninflammatory disease, the evidence provided by the authors contradicts the previous assumption that PPRD is noninflammatory.

In a larger cohort study Romano et al., the authors genetically characterized 29 pediatric patients with arteriovenous cerebral high-flow shunts. Of the 29 patients, 11 carried variants in genes associated with vascular function, 5 received a genetic diagnosis, 1 child had a variant of uncertain significance in the EPHB4 gene, and 5 reported variants in novel genes probably linked to cerebrovascular disorders. Interestingly, the authors revealed that genotype-phenotype correlations can have significant implications for pathophysiological mechanisms, therapeutic approaches, and outcomes. Accordingly, variants in all known genes associated with arteriovenous cerebral shunts were reported in VGAM patients, whereas cutaneous angiomas were specific to RASA1 mutations.

Lastly, to improve understanding of these diseases, provide insights into early diagnosis and intervention, and support stratified management, Chen et al. analyzed the clinical characteristics and genetic features of children with adverse reactions to the *Bacillus* Calmette-Guérin (BCG) vaccine. Overall, the study provides a comprehensive clinical and genetic characterization of pediatric adverse reactions to the BCG vaccine over 10 years. By distinguishing between localized BCG-itis and disseminated BCG disease, and further stratifying patients according to underlying primary immunodeficiency, the authors highlight important clinical and immunological markers associated with disease severity and prognosis. The findings underscore the strong association between disseminated BCG disease and severe immune dysfunction, particularly severe combined immunodeficiency, which was linked to markedly higher mortality. Moreover, the authors emphasized the importance of early recognition of warning signs such as fever, hepatosplenomegaly, elevated inflammatory markers, and abnormal immune profiles to facilitate prompt diagnosis and tailored management. Overall, this work contributes valuable evidence supporting early immunological screening and genetic evaluation in infants with BCG vaccine complications, with significant implications for improving outcomes through timely intervention.

Collectively, these contributions emphasize the importance of early immunological screening in infants with clinical complications, as well as the diagnostic value of integrating phenotypic and molecular analyses in patients with suspected Inborn Errors of Immunity.

